# Probiotics, prebiotics, and synbiotics for the treatment of dementia: Protocol for a systematic review

**DOI:** 10.1097/MD.0000000000018608

**Published:** 2020-01-31

**Authors:** Wenyuan Li, Jing Guo, Yifeng Shen, Ling Huang, Bingshuang Leng, Dong Fan, Liyao Shui, Chongli Chen

**Affiliations:** a Hospital of Chengdu University of Traditional Chinese Medicine, Chengdu, Sichuan Province; b Beijing University of Chinese Medicine, Beijing, P.R. China.

**Keywords:** dementia, prebiotics, probiotics, protocol, synbiotics, systematic review

## Abstract

**Background:**

The number of dementia patients in the world is large, and the number of dementia patients will continue to rise in the future, which will bring a heavy social and economic burden. No interventions have been found to cure dementia. Medication can delay the progression of the disease and impose an economic burden. Some non-drug therapies often require the care of the caregiver. Probiotics, prebiotics, and synbiotics may intervene in dementia through microbiota-gut-brain axis (MGBA). However, their effectiveness and safety are still obscure and deserve further investigation. The purpose of this study is to assess the effect and safety of probiotics, prebiotics, and synbiotics in treating dementia.

**Methods:**

We will summarize and meta-analyze randomized controlled trials (RCTs) of probiotics, prebiotics, and synbiotics for the treatment of dementia. RCTs comparing probiotics, prebiotics, and synbiotics with blank control, placebo or conventional therapies will be included. RCTs comparing probiotics, prebiotics, and synbiotics plus conventional therapies with conventional therapies alone will also be included. The following electronic databases will be searched: PubMed, Cochrane Library, EMBASE, CNKI, CBM, VIP, and WAN FANG DATA. The methodological quality of RCTs will be assessed using the Cochrane risk assessment tool. All trials included will be analyzed according to the criteria of the Cochrane Handbook. Review Manager 5.3, R-3.5.1 software will be used for publication bias analysis. Grading of Recommendations Assessment, Development and Evaluation (GRADE) pro-GDT web solution will be used for evidence evaluation.

**Results:**

This review will evaluate the effects of probiotics, prebiotics, and synbiotics on cognitive function, behavioral and psychological symptoms of dementia, quality of life (QOL), functional performance in activities of daily living, and compliance with the intervention and safety in patients with dementia.

**Conclusions:**

This review will provide clear evidence to assess the effectiveness and safety of probiotics, prebiotics, and synbiotics for dementia.

**OSF registration number**: DOI 10.17605/OSF.IO/2Q3AK.

## 1 Introduction

Dementia is a syndrome characterized by a decline in memory, thinking, behavior, and daily activities. There are about 50 million people with dementia in the world, and 10 million new cases each year. Dementia has physical, psychological, social, and economic effects on the patients themselves, as well as on their caregivers, families, and society as a whole. In 2015, the global cost of dementia to society was estimated at $818 billion, or 1.1% of global gross domestic product. It is predicted that the total number of people with dementia will reach 82 million by 2030 and reach 152 million by 2050, most of which can be attributed to an increase in the number of people with dementia living in low- and middle-income countries.^[1]^

Dementia can usually be divided into 5 types: Alzheimer disease (AD), vascular dementia (VD), dementia with Lewy bodies (DLB), dementia in Parkinson disease (PDD), and frontotemporal dementia (FTD). In at least a quarter of patients, different subtypes exist simultaneously.^[2,3]^

No interventions have been found to cure dementia. Current medical treatment can only delay the development of symptoms in patients with dementia but has no effect on the survival of patients.^[4,5]^ In view of this situation, some people are beginning to explore polypharmacy, including supplements, in search of relatively inexpensive and more effective palliative treatments.^[6]^ At the same time, various non-drug therapies have also been used in dementia patients and their caregivers to improve various symptoms of dementia, and relevant evidence has emerged.^[7–10]^

Intestinal flora is closely related to nervous system diseases. For instance, lower *Bififidobacterium* and/or *Lactobacillus* counts were observed in subjects with major depressive disorder.^[11–13]^ In addition, the number of *Bififidobacterium* in the gut microbiota of patients with AD is lower than that of normal people.^[14]^ The evidence of microbiota-gut-brain axis (MGBA) communication can be found from the relationship between gut dysbiosis with functional gastrointestinal disorders and central nervous disorders.^[15]^ Interventions through MGBA may be a choice for nervous system diseases, including dementia.

Probiotics are a class of active microorganisms that are beneficial to the host by colonization in the human body and altering the composition of the flora at a certain part of the host. Prebiotics are non-digestible food ingredients that have a beneficial effect on the host by selectively stimulating the growth and activity of probiotics to improve host health. Synbiotics are a combination of probiotics and prebiotics.^[16,17]^ Probiotics, prebiotics, and synbiotics may regulate the neurotransmitters and proteins, including gamma-aminobutyric acid (GABA), serotonin, glutamate, and brain-derived neurotrophic factor (BDNF), which play important roles in controlling the neural excitatory-inhibitory balance, mood, cognitive functions, learning and memory processes.^[18–20]^ Several probiotics strains were reported as effective for neuropsychological symptoms from animal studies^[21,22]^. However, the evidence is not consistent in the treatment of AD with probiotics.^[23]^ This systematic review will provide valuable evidence to identify effective intervention approaches.

## 2 Methods

This systematic review has been registered in OSF (https://osf.io/2q3ak), registration number: DOI 10.17605/OSF.IO/2Q3AK.

We will develop and report this study in compliance with the Preferred Reporting Items for Systematic Reviews and Meta-Analyses (PRISMA).^[24]^ The procedure of this protocol was based on Preferred Reporting Items for Systematic Review and Meta-Analysis Protocols (PRISMA-P) guidance.^[25]^

### 2.1 Database search

Three English medical databases (Cochrane Library, PubMed, and EMBASE) and 4 Chinese medical databases (China National Knowledge Infrastructure Database [CNKI], Chinese Biomedical Literature Database [CBM], VIP Chinese Science and Technology Periodical Database [VIP], and Wan Fang Data) will be systematically searched from their inceptions up to July 31, 2019. The search strategy will be based on the guidance of the Cochrane handbook.^[26]^ The search formulas of the databases are adjusted according to the following forms: (probiotic OR prebiotic OR synbiotic OR Bifidobacter∗ OR Lactobacill∗ OR Saccharomyce∗ OR Lactic Acid Bacteria) AND (dementia OR Alzheimer disease) AND (random∗). All relevant publications including academic dissertation and conference will be researched. There will be no language and publication date restrictions.

### 2.2 Inclusion criteria

#### 2.2.1 Types of studies

Only randomized controlled trials (RCTs) will be included.

#### 2.2.2 Types of participants

All of the participants were diagnosed as dementia.

#### 2.2.3 Types of interventions

Probiotics, prebiotics, or synbiotics as the intervention treatment compared with blank control, placebo, or conventional treatment will be selected. Probiotics, prebiotics, or synbiotics in combination with conventional therapies compared with conventional therapies alone will also be included.

#### 2.2.4 Types of outcome measures

Primary outcomes: cognitive function (measured by validated scales); behavioral and psychological symptoms of dementia (measured by validated scales). Secondary outcomes: quality of life (QOL); functional performance in activities of daily living; compliance with the intervention; adverse event.

### 2.3 Exclusion criteria

(1) The unrelated and duplicated documents will be deleted.
(2) Animal experiments, reviews, theoretical discussions, experience summaries, and case reports.
(3) Review articles without original data.

### 2.4 Data collection and extraction

Referring to the Cochrane collaborative network system evaluator handbook^[26]^: importing the search results into the document management software of NoteExpress (version:3.2, Beijing Aegean Software Company, Beijing, China); excluding the duplicate literature using NoteExpress3.2 and excluding the unrelated articles by reading the title and abstract; reading the full text and reserving clinical studies that meet the inclusion criteria. Two researchers (WYL and JG) will extract the data independently using a self-developed data extraction form. The differences encountered in the process will be resolved by discussing with another team member (CLC), to determine, by agreement, the final selection of studies.

**Figure 1:**
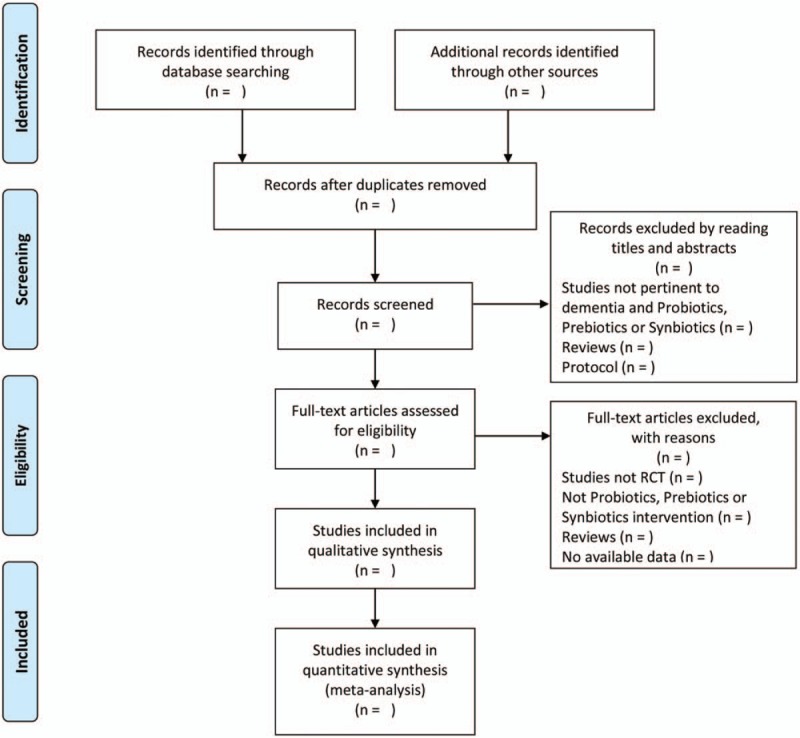
Flow diagram of the study selection process. Arrows = flow directions or reasons for exclusion of trials; RCT = randomized controlled trial.

Data extraction contents will include: general information: research ID, author, title, publication status, report sources and fund support. Methodology information: design, number of arms, random sequence generation, allocation concealment, blinding, incomplete outcome data, selective reporting, sample size calculation and baseline comparability. Participant information: diagnostic criteria, inclusion criteria, exclusion criteria, setting, population, sample size, age, sex, and course of disease. Intervention information: name of intervention and comparison, dosage form, comparison, duration of treatment, and patient follow-up. Outcomes. Adverse events. The selection process was shown in a PRISMA flow chart (http://www.prisma-statement.org/) (Fig. 1).

### 2.5 Assessment of methodological quality

Risk of bias will be assessed by Cochrane risk assessment tool^[26]^ in 7 domains: random sequence generation, allocation concealment, blinding of the participants and personnel, blinding of outcomes assessment, incomplete outcome data, selective outcome reporting, and other bias. These domains will classify “low risk” if adequate, “high risk” if not adequate, and “Unclear” if not well described by the authors in such a way that its adequacy is describable.

The two researchers (WYL and JG) will independently assess the risk of bias for each included study. We will use “L,” “H,” and “U” as a code for the evaluations of the above bias risks. “L” indicating a low risk of bias, “H” indicating a high risk of bias, “U” indicating that the risk of bias is unclear. Disagreements will be resolved by discussion between all the researchers. When necessary, we will contact the study authors to inquire some missing information. Trials of high risk of bias will be considered when conducting sensitivity analysis.

### 2.6 Data synthesis and analysis

Review Manager Software (RevMan, Version 5.3 for windows, The Cochrane Collaboration, Oxford, England) will be used to analyze and synthesize the outcomes.

Quantitative synthesis will be done when clinical heterogeneity is not considered by at least 2 authors in discussion. Continuous variables will be described by mean difference (MD), *P-*value, and 95% confidence interval (CI). For dichotomous outcomes, we will use the relative risk (RR), with 95% CI and *P* values, to evaluate the efficacy and safety of probiotics/prebiotics/synbiotics. *I*^2^ test will be used to judge the heterogeneity of meta-analysis. *I*^2^ value >50% or more will be considered as an indication of substantial heterogeneity. If heterogeneity exists in the pooled studies, the data will be analyzed using a random effects model. Otherwise, a fixed effect model will be adopted. Sensitivity analysis or subgroup analysis will be performed if included trials are sufficient. The grouping factor for subgroup analysis will be type of dementia, dementia severity, and treatment duration. Qualitative description will be adopted if clinical heterogeneity exists.

### 2.7 Publication bias

The publication bias will be analyzed by the Egger test. The analysis software is R 3.5.1 for Windows.

### 2.8 Quality of evidence

This study evaluates the evidence according to Grading of Recommendations Assessment, Development and Evaluation (GRADE) standard, which refers to grading of recommendations assessment, development and evaluation. GRADE Pro GDT online software will be used to form the summary of findings table (SoF table).

## 3 Discussion

The number of microorganisms in the human gut is 100 times greater than that of human cells.^[27,28]^ Gut microbiota has been reported to be involved in various physiological processes, including immunomodulation, energy balance, and activation of the enteric nervous system (ENS).^[29–33]^ The above facts provide an idea for intervening in dementia by regulating intestinal flora.

Dementia is the most common type of neurodegenerative disease with increasing socioeconomic burden.^[34]^ Probiotics, prebiotics, and synbiotics are cheaper than medical treatments and are easier to handle than other non-drug therapies, reducing the burden on caregivers. For patients from low and middle-income families who lack care, they are more advantageous.

We need to obtain systematic evidence to prove the effectiveness and safety of probiotics/prebiotics/synbiotics. We also need to assess the applicability of probiotics to patients with different severity of dementia. This study will solve the above problems.

## Author contributions

Wenyuan Li, Jing Guo, and Chongli Chen conceived and designed the project.

Yifeng Shen, Ling Huang, Bingshuang Leng, Dong Fan, and Liyao Shui implemented the methods.

Wenyuan Li and Jing Guo contributed analysis tools and edited review.

Chongli Chen revised and supervised the manuscript.

All authors read and approved the final manuscript.

Wenyuan Li orcid: 0000-0003-2914-684X.

Jing Guo orcid: 0000-0001-9861-0250.

Yifeng Shen orcid: 0000-0003-0356-1420.

Ling Huang orcid: 000-0002-0887-5544.

Chongli Chen orcid: 0000-0002-0189-3301.
